# Quantitative Genetics of Food Intake in *Drosophila melanogaster*

**DOI:** 10.1371/journal.pone.0138129

**Published:** 2015-09-16

**Authors:** Megan E. Garlapow, Wen Huang, Michael T. Yarboro, Kara R. Peterson, Trudy F. C. Mackay

**Affiliations:** 1 Program in Genetics, North Carolina State University, Raleigh, NC, 27695–7614, United States of America; 2 Department of Biological Sciences, North Carolina State University, Raleigh, NC, 27695, United States of America; 3 W. M. Keck Center for Behavioral Biology, North Carolina State University, Raleigh, NC, 27695, United States of America; Duke University, UNITED STATES

## Abstract

Food intake is an essential animal activity, regulated by neural circuits that motivate food localization, evaluate nutritional content and acceptance or rejection responses through the gustatory system, and regulate neuroendocrine feedback loops that maintain energy homeostasis. Excess food consumption in people is associated with obesity and metabolic and cardiovascular disorders. However, little is known about the genetic basis of natural variation in food consumption. To gain insights in evolutionarily conserved genetic principles that regulate food intake, we took advantage of a model system, *Drosophila melanogaster*, in which food intake, environmental conditions and genetic background can be controlled precisely. We quantified variation in food intake among 182 inbred, sequenced lines of the *Drosophila melanogaster* Genetic Reference Panel (DGRP). We found significant genetic variation in the mean and within-line environmental variance of food consumption and observed sexual dimorphism and genetic variation in sexual dimorphism for both food intake traits (mean and variance). We performed genome wide association (GWA) analyses for mean food intake and environmental variance of food intake (using the coefficient of environmental variation, *CV*_*E*_, as the metric for environmental variance) and identified molecular polymorphisms associated with both traits. Validation experiments using RNAi-knockdown confirmed 24 of 31 (77%) candidate genes affecting food intake and/or variance of food intake, and a test cross between selected DGRP lines confirmed a SNP affecting mean food intake identified in the GWA analysis. The majority of the validated candidate genes were novel with respect to feeding behavior, and many had mammalian orthologs implicated in metabolic diseases.

## Introduction

Food intake is a fundamental fitness trait as it is essential for the maintenance of energy balance and survival. In humans, consumption of excessive calories is associated with an increased incidence of type 2 diabetes, obesity, cardiovascular disease, and other disorders and diseases [1–3]; while insufficient caloric intake is correlated with abnormal liver function and skin and other disorders [4, 5]. However, dissecting the genetic and environmental contributions to variation in food intake in human populations is challenging, due to difficulties in quantifying food intake [6–13] and small effects of segregating variants that together account for only a small fraction of the estimated genetic variance [14–18]. Using model organisms such as *Drosophila melanogaster* to delineate the genetic and neural basis of food consumption and metabolism, obesity, type 2 diabetes, and other traits and disorders connected with food consumption can identify evolutionarily conserved candidate genes and pathways relevant to human biology [19, 20].

The development of rapid and reproducible techniques for quantifying food intake has facilitated many recent studies on the genetic, neural, and environmental factors modulating food consumption in *D*. *melanogaster*. Measuring feeding frequency by quantifying proboscis extension provides an estimate of total food intake, and can be applied to the same group of flies over time [21] and automated for continuous resolution [22]. Radioactive tracers can permit measurement of food intake and fecal and egg excretion [23], while the development of the Capillary Feeding (CAFE) assay allows for continuous measurement of food intake with high resolution [24, 25].

The genetic and environmental basis of food consumption in *Drosophila* has many components. *Drosophila* utilize gustatory receptors (GRs), olfactory receptors (ORs) and odorant binding proteins (OBPs) to sense food and assess its quality as they integrate food cues with physiological state to affect feeding decisions. Expression of short neuropeptide F (sNPF) and its receptor in OR42b neurons is required for increasing food search behavior induced by starvation [26]. Food odors elicit rapid, transient physiological and transcriptional increases in circulating glucose and expression of four of the eight *Drosophila* Insulin-Like Peptides (DILPs) and adipokinetic hormone (AKT) [27]. OBPs are thought to transport bitter hydrophobic tastants to gustatory receptors to modulate food intake [28]. The receptor for trehalose, Gr5a, acts with the G-protein alpha subunit, Gsα, to allow sugar taste transduction [29]. Gustatory perception of food quality can distinguish among major tastant groups but not individual compounds within a modality [30]. Gustatory sugar acceptance is modulated by dopaminergic signaling [31]. Acceptance of unpalatable, often bitter, foods increases with long-term exposure, with attenuation of gustation achieved through activation of Transient Receptor Potential-Like (TRPL) localized in the GR neurons [32]. In the brain, Gr43a senses fructose circulating in the hemolymph, promotes feeding in hungry flies, and suppresses feeding in satiated flies [33]. Integration of OBPs, GRs, and subsequent neuroendocrine and motor responses can be modulated by gustatory interneurons connecting food sensing modalities with neuroendocrine and motor outputs [34].

Gustatory and olfactory sensing assess the tastants and odorants of food, while the brain assesses caloric content independent of gustatory and olfactory modalities. The brain directly senses caloric content of circulating sugar without tasting sugar via the hemolymph, and communicates necessary feeding and metabolic changes [35]. In fact, caloric sensing by the brain forms metabolic memories that allow *Drosophila* to balance caloric intake with metabolic state independent of taste [36]. Adult *D*. *melanogaster* increase their feeding rate under dietary restriction, thereby adjusting caloric intake to physiological needs [37]. Odorant sensing to locate and anticipate feeding, gustation to assess palatability, and caloric density assessment by the brain independent of taste are integrated with physiological state to regulate metabolism and determine feeding behavior.

The central nervous system serves as the master regulator of food intake, integrating various physiological signals with external cues. Decreases in food intake are achieved through various peptides and signaling modalities: Leucokinin (Lk) and the Leucokinin Receptor (Lkr) [38], Forkhead Box O transcription factor (FOXO) [39–41], Allatostatins [42], serotonin [43], neuronal populations of *c673a* [44], peripheral cAMP-responsive transcription factor (CREB) [45], hugin [46–48], Rac2 [49], and Unpaired 2 (Upd2) [50] all elicit decreased food intake. In comparison, there are fewer signaling peptides involved in increasing food intake: SP [51], sNPF [52–54], and dopamine [55] elicit feeding increases. Increases and decreases of food intake are further modulated by a complex coordination of neuronal signaling. A single pair of Feeding neurons control the initiation of food intake [56], and four GABAergic interneurons promote the cessation of feeding in response to satiety and gustatory cues [57]. Insulin, serotonergic, octopaminergic, GABAergic, and dopaminergic signaling pathways all act and interact to orchestrate physiological and metabolic cues, sensory input, and signaling peptides into the appropriate feeding responses [31, 43, 49, 57–61]. The decision to eat is accompanied by extension of the proboscis and fluid ingestion by motor neuron-driven pumping; these neurons exhibit prolonged activation to palatable tastants [62].

Despite these recent advances in understanding the integration of environmental, physiological, and neuroendocrine factors affecting initiation, continuation, and cessation of food intake, the extent to which these genes and pathways affect natural variation in food intake in *Drosophila* is unknown. Here, we used the inbred, sequenced lines of the *D*. *melanogaster* Genetic Reference Panel (DGRP) [63, 64] to quantify substantial genetic variation in both mean food consumption and the within-line environmental variance (measured using the coefficient of environmental variation, *CV*_*E*_) of food consumption [65–71]. We performed genome wide association (GWA) analyses for both traits and identified novel candidate genes, many of which we functionally validated using single nucleotide polymorphisms (SNPs) in the DGRP [72] or RNAi alleles.

## Results

### Quantitative genetics of mean food consumption in the DGRP

**Fig 1:**
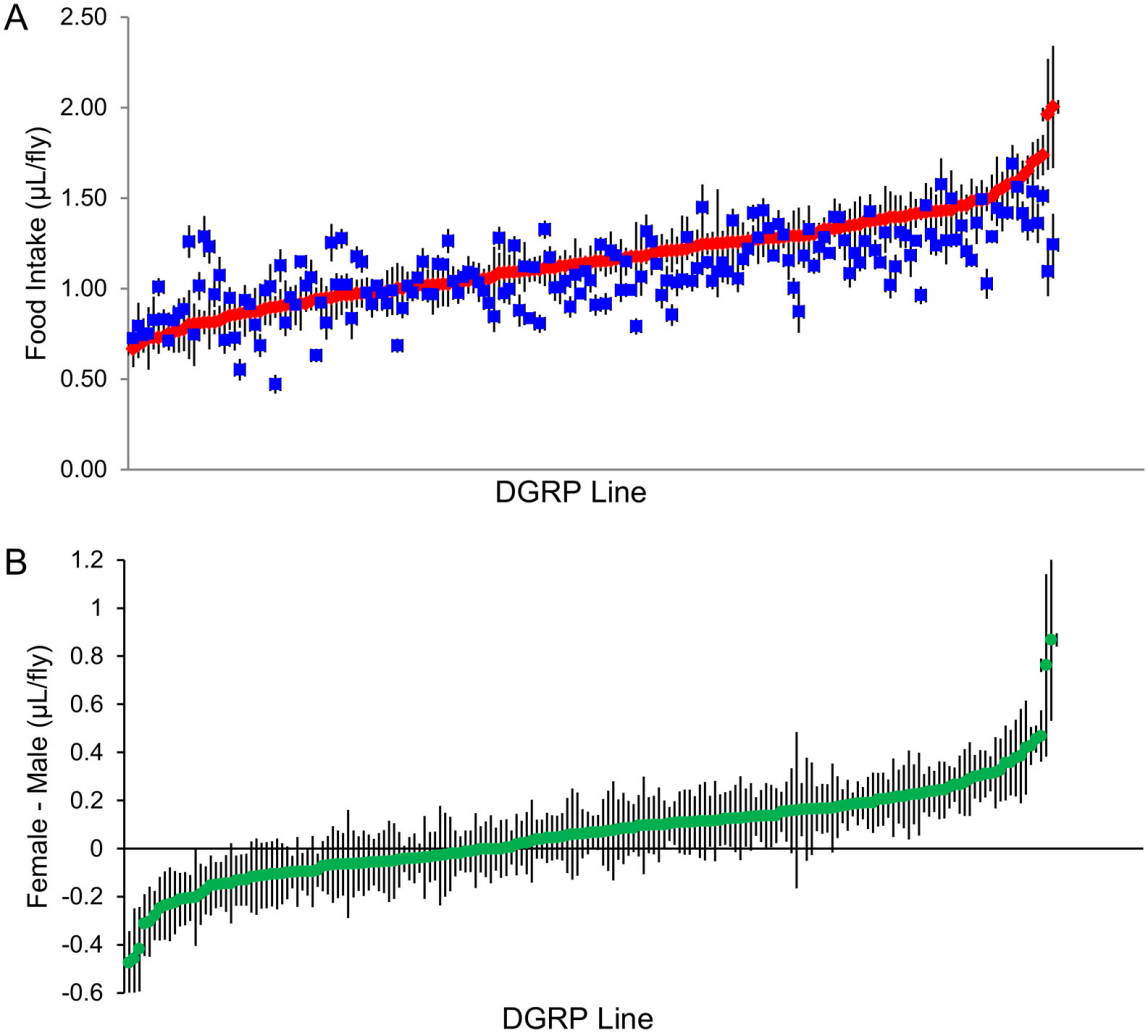
Variation in mean food intake in the DGRP. (**A**) Mean food intake in 182 DGRP lines, arranged in order from lowest to highest in females (red diamonds). Mean food intake of males (blue squares) are given in the same order as the female scores. (**B**) Sexual dimorphism of food intake. The female-male difference in mean food intake of each line is ordered from lowest to highest. All error bars are ± SE.

We assessed total consumption of 4% sucrose over 24 hours for 182 DGRP lines (S1 Table) using a modified version of the CAFE assay [24]. We found substantial genetic variation in food consumption among the DGRP lines, with a broad sense heritability of *H* ^*2*^ = 0.45 (Fig 1A, Table 1). Food intake is sexually dimorphic (*P* < 0.0001 for the sex term), with females generally consuming more than males. In addition, there is genetic variation among the DGRP in the magnitude of sexual dimorphism in food consumption (Fig 1B, Table 1, *P* < 0.0001 for the sex by line interaction term), with a cross-sex genetic correlation of *r*_*GS*_ = 0.68. Thus, we expect the genetic architecture of mean food intake to be at least partially sex-specific.

**Table 1:** Analyses of variance (ANOVA) of food intake.

Analysis	Source of Variation	df	MS	F	*P*-value	*σ* ^2^ (SE)
Sexes	Sex	1	423.60	33.43	<0.0001	Fixed
Pooled	Line	183	125.29	9.89	<0.0001	8.21 (1.09)
	Sex x Line	181	24.43	1.93	<0.0001	1.92 (0.42)
	Error	1867	12.37			12.66 (0.41)
Females	Line	182	86.15	5.35	<0.0001	11.42 (1.48)
	Error	938	16.09			16.09 (0.74)
Males	Line	182	63.62	6.90	<0.0001	8.91 (1.10)
	Error	929	9.22			9.21 (0.43)

df: degrees of freedom; MS: type III mean squares; F: F-statistic; *σ* ^2^ variance component estimated using restricted maximum likelihood.

Many other physiological and life history traits have been measured in the DGRP lines, enabling us to estimate genetic correlations between these traits and food intake. In total, we tested correlations with food intake for 33 traits in females and 66 traits in males. The most significant correlation with food intake was a negative correlation with starvation resistance in both sexes (*r* = -0.31, *P* < 0.0001 for males and females) [64]. We observed nominally significant and modest correlations between food intake and several sleep traits and lifespan in both sexes [67, 73]; resistance to oxidative stress induced by paraquat [74] and menadione sodium bisulfite (MSB) in females [75]; and several nutritional index values in males [76] (S2 Table). None of the correlations between food intake and body weight or aspects of metabolism [77] approached significance, suggesting that consumption is independent of size.

### Quantitative genetics of within-line variation in food consumption in the DGRP

Several studies have shown that the within-line environmental variance of inbred lines is heritable [65–69, 71]. We used Brown-Forsythe and Levene’s tests to assess whether there was heterogeneity of within-line environmental variance in food consumption among the DGRP lines. These tests are modifications of one-way ANOVA that use, respectively, the absolute deviation of each data point from the line median and mean as the response variable. Indeed, we find highly significant heterogeneity of environmental variance among the DGRP lines (Table 2), suggesting heritability of this trait. Within some inbred DGRP lines, individuals consume relatively constant amounts of food, whereas within others, individuals consume widely varying amounts of food.

**Table 2:** Brown-Forsythe and Levene’s tests for unequal variance.

Test	Sex	df	F	*P*-value
Brown-	Male	181	1.2246	0.0337
Forsythe	Female	182	1.4663	0.0002
Levene	Male	181	1.6288	<.0001
	Female	182	2.0492	<.0001

df: degrees of freedom; F: F statistic.

**Fig 2:**
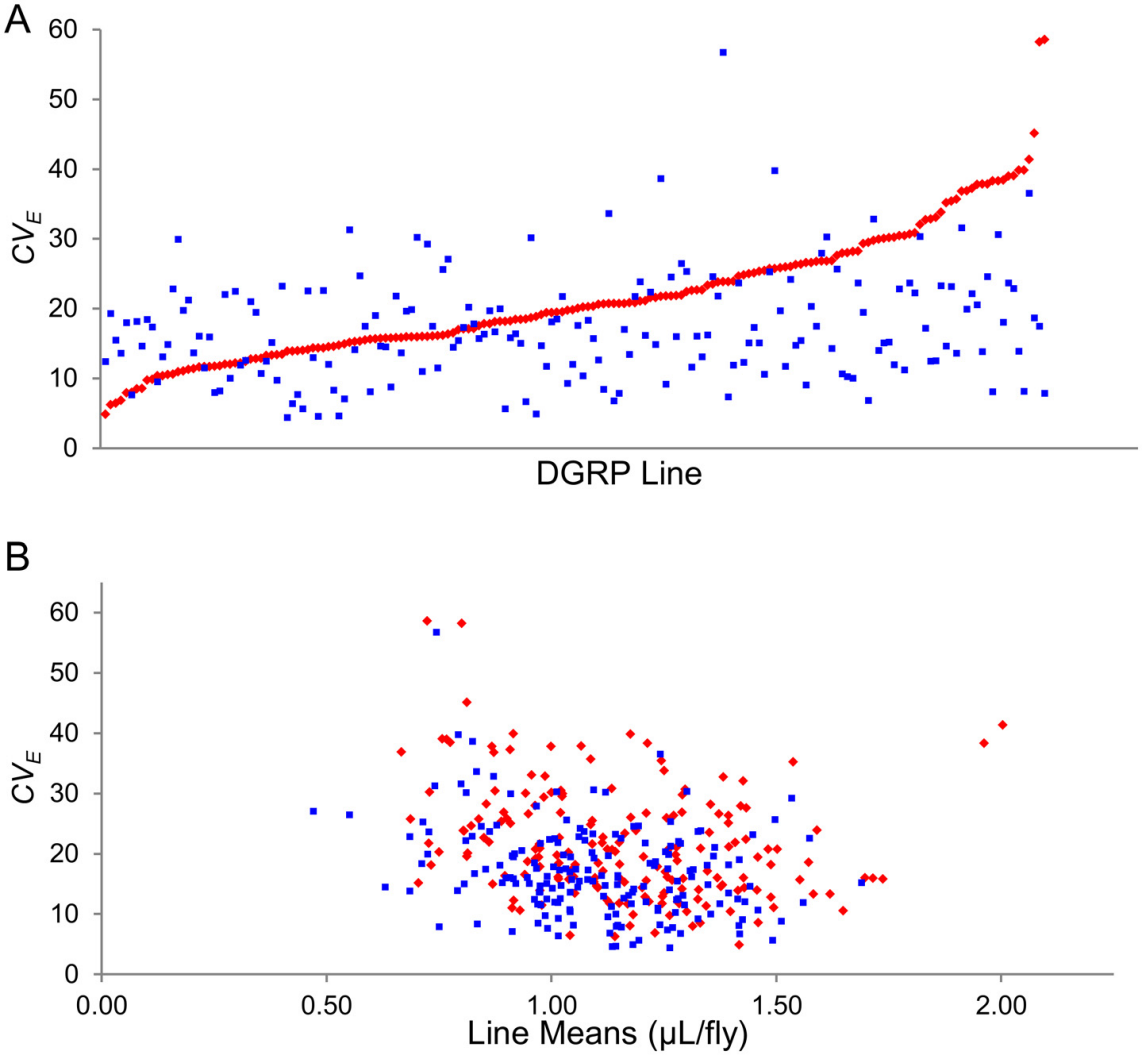
Variation in *CV*_*E*_ of food intake in the DGRP. (**A**) *CV*_*E*_ of food intake, arranged in order from lowest to highest in females (red diamonds). *CV*_*E*_ of food intake of males (blue squares) are given in the same order as the females. (**B**) Association of mean and *CV*_*E*_ for food intake for females (red diamonds) and males (blue squares).

We used the coefficient of within-line variation (*CV*_*E*_) as the metric for within-line environmental variance (Fig 2). The correlation of *CV*_*E*_ between males and females is *r* = 0.16, *P* = 0.031 (Fig 2). The approximate 95% confidence limits of this correlation are 0.012–0.308; thus the correlation is barely significantly different from zero and clearly different from unity, suggesting largely sex-specific genetic architecture of variation in environmental variance. The correlation between mean and *CV*_*E*_ in food intake is significantly different from zero, but small and negative (*r* = -0.27, *P* = 0.0002 for females; and *r* = -0.29, *P* < 0.0001 for males). Therefore, only 7–8% of the total variance in *CV*_*E*_ for food consumption can be explained by the association with the mean. We assessed whether variance in *CV*_*E*_ could be a consequence of variation in the numbers of segregating sites in the DGRP lines [64], as it has been hypothesized that within line environmental variation may be inversely related to heterozygosity [78, 79]. This hypothesis was not supported by these data: the correlation between *CV*_*E*_ and the number of segregating sites was not significant for females (*r* = 0.026, *P* = 0.72) or males (*r* = -0.106, *P* = 0.16; S1 Fig).

### GWA analyses of food consumption

We performed GWA analyses for mean and *CV*_*E*_ of food intake using 1,920,276 polymorphic variants with minor allele frequencies ≥ 0.05. We performed four analyses for each trait: the average of females and males, the difference between females and males (formally equivalent to the sex by line interaction), and for females and males separately. Prior to performing the GWA analyses, we tested and corrected for any effects of *Wolbachia* infection and common polymorphic inversions as well as polygenic relatedness [64]. For mean food intake, we observed a significant effect of *Wolbachia* in females (*P* = 0.03) and for the difference between females and males (*P* = 0.026); a significant effect of *In(3R)K* for males (*P* = 0.05); and a significant effect of *In(3R)Mo* for males (*P* = 0.04) and for the difference between females and males (*P* = 0.04) (S3 Table). None of these covariates was significant for *CV*_*E*_ of food intake (S3 Table).

At a nominal reporting threshold of *P* < 10^−5^, we found 74 variants in or near (± 1 kb) 54 genes affecting mean food intake (S4 Table) and 160 variants in or near 101 genes affecting food intake *CV*_*E*_ (S5 Table). Two genes, *Dystrophin* (*Dys*) and *CG1136*, were associated with both the mean and *CV*_*E*_. None of the variants were significant following a Bonferroni correction (*P* = 2.6 × 10^−8^) for multiple tests. However, 12 of the 153 genes implicated in these analyses have been previously associated with aspects of feeding (S6 Table). Among the candidate genes for mean food intake, *retained* (*retn*), *CG10477* and *CG42788* were previously associated with adult amino acid consumption from gene expression and GWA analyses in the DGRP [80], and *Aldolase* (*Ald*) was associated with adult transcriptional response to dietary restriction [81] (S6 Table). Among the candidate genes for *CV*_*E*_ of food intake, previous associations have been reported for *Ecdysone-inducible gene L1* (*ImpL1*) with larval sugar feeding [82]; *CG3502*, *frizzled* (*fz*) and *Zwilch* with amino acid intake [80]; *Octopamine β3 receptor* (*Octβ3R*) with larval hunger-driven feeding [83]; *Organic anion transporting polypeptide 74D* (*Oatp74D*) with high glucose rearing [76]; *pointed* (*pnt*) with larval nutritient control of mitochondrial biogenesis [84]; and *Tyramine β hydroxylase* (*Tbh*) with adult sweet taste-induced memory formation [85] and adult response to sucrose concentration [86] (S6 Table).

We used gene ontology (GO) enrichment analyses [87, 88] to place the top associations in context. None of the GO terms for either trait had enrichment scores passing a 5% false discovery rate (S7 Table). At nominal *P*-values < 0.05, the top genes associated with mean food intake were enriched for olfactory and chemosensory behavior, and epidermal growth factor (EGF) signaling (S7 Table). The top genes associated with *CV*_*E*_ of food intake were associated with behaviors, G-protein coupled signaling, and regulation of transcription (S7 Table).

### SNP-based functional validation

**Fig 3:**
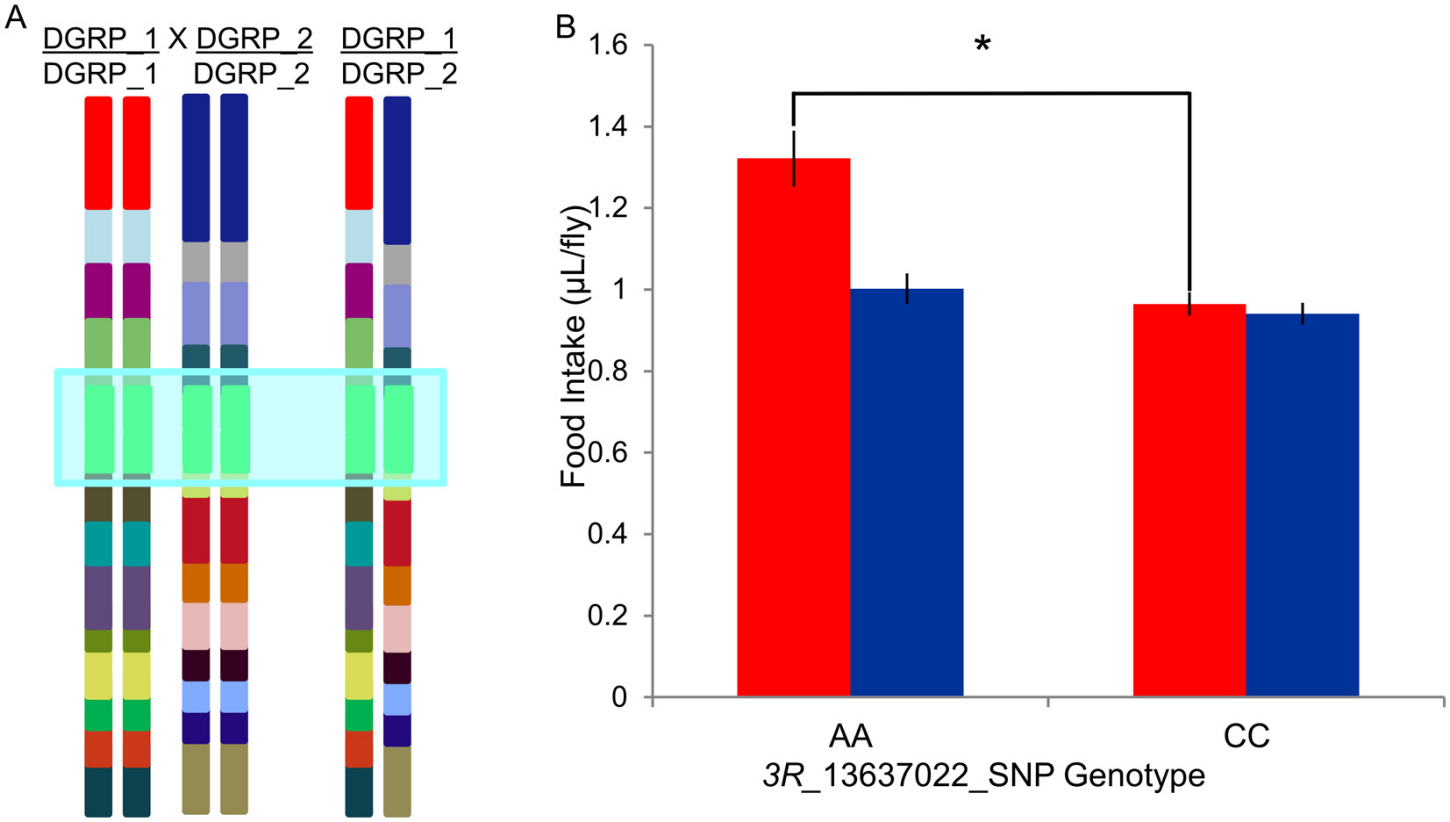
SNP-based functional validation. **(A)** Mating scheme for generating outbred lines homozygous for major and minor SNP alleles. Two parental genotypes homozygous for the focal highlighted SNP and the tested F_1_ genotype are depicted. **(B)** *3R*_13637022_SNP validation results for females (red bars) and males (blue bars) (± SE). *: *P* = 0.029.

In order to assess whether a putative regulatory SNP affects feeding behavior, we chose one SNP, *3R*_13637022_SNP, an A/C polymorphism 174 bp upstream of *CG18012* and 241 upstream of *tincar* with a female-specific effect on food intake (female *P*-value = 5.14 × 10^−6^; male *P*-value = 2.11 × 10^−4^, S2 Table) to functionally validate in outbred genetic backgrounds. We selected this SNP based on its *P*-value and a minimum of ten minor-allele-containing DGRP lines that we had measured. We created five different F1 genotypes from 10 DGRP lines homozygous for the minor allele (C) and five F1 genotypes from 10 DGRP lines homozygous for the major allele (A), and measured their food intake (Fig 3A, S8 Table). These lines have randomized genetic backgrounds with the exception of the target polymorphism. We indeed confirmed a significant (*P* = 0.029; Fig 3B) female-specific effect of this polymorphism on food consumption in the predicted direction from the GWA analysis, with the major allele associated with an increase in food intake. However, the magnitude of this effect (0.36 *μ*L), was not as large as the effect from the GWA analysis (1.37 *μ*L). This discrepancy of effect size can be attributed to the Beavis Effect [89, 90], whereby the effect sizes from GWA analyses are overestimated as the predicted effects are generated from quantitative trait loci (QTL) that reach a critical, predetermined threshold; the distribution of QTL from which effect size is predicted is, therefore, truncated, creating an upward bias in predicted effects [89–91].

### RNAi functional validation

**Fig 4:**
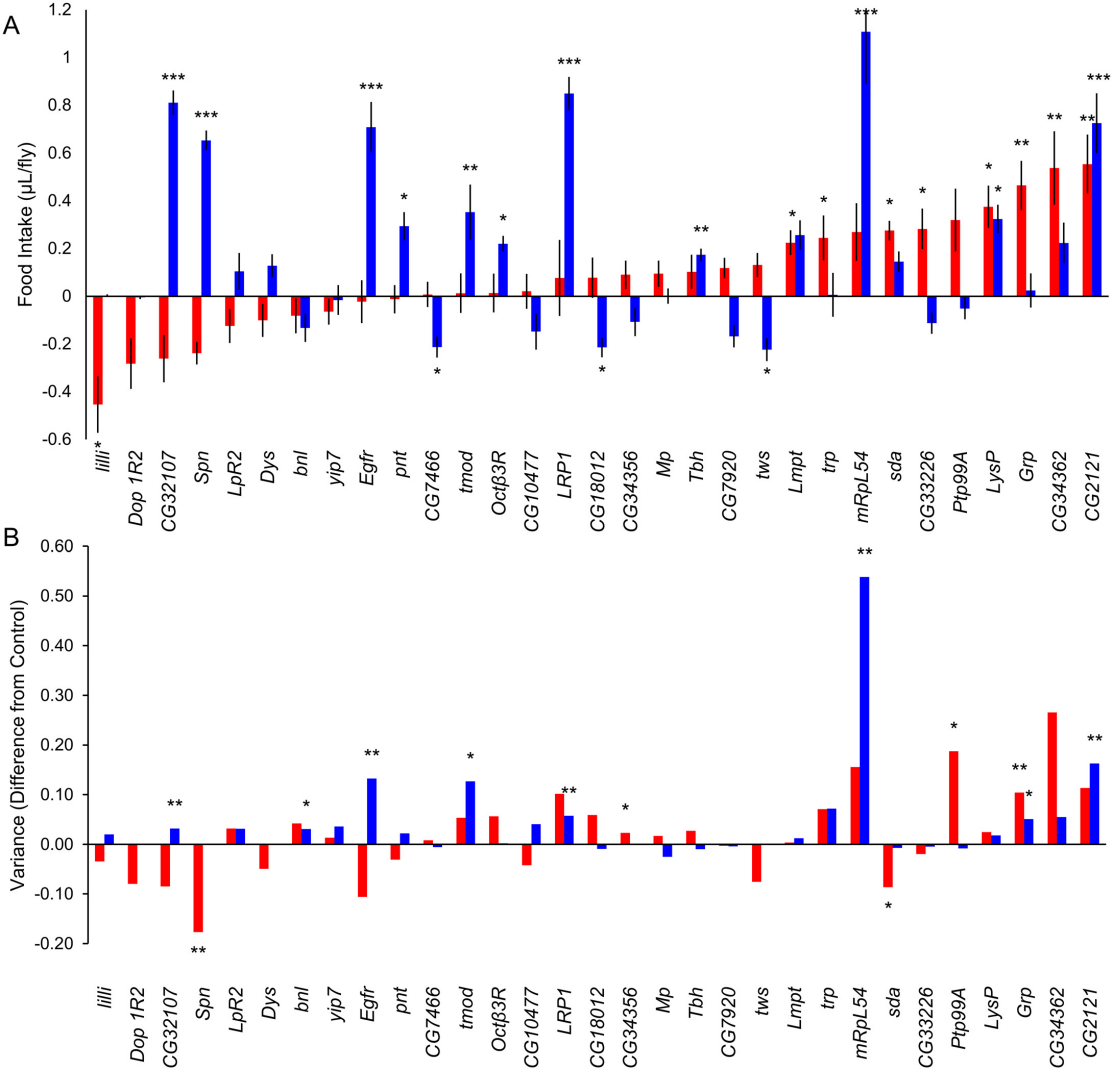
RNAi functional validation. Effects of RNAi knock down on (**A**) mean food intake and (**B**) within-genotype variance of food intake of females (red bars) and males (blue bars) for 31 candidate genes. All values are deviations from the control. Error bars are ± SE. *: *P* < 0.05; **: *P* < 0.01; ***: *P* < 0.0001.

We selected 31 genes for functional validation using RNAi knockdown and a weak, ubiquitous *GAL4* driver, *Ubi156*-*GAL4*. We chose these genes based on the availability of RNAi lines, patterns of adult gene expression in *Drosophila* (ubiquitous, or in gut, fat body, and/or brain), the existence of a human ortholog (S9 Table) and/or involvement in feeding-related processes (S6 Table). The weaker *Ubi156*-*GAL4* driver line was used to prevent lethality often observed with stronger ubiquitin promoters, while still allowing ubiquitous expression. Although the top candidate genes from the mean and *CV*_*E*_ GWA analyses are largely distinct, this is a consequence of using *CV*_*E*_ as the metric for the within-line variance, as it ameliorates the dependency of the variance on the mean. However, there is a real relationship between mean and within-line variance of food consumption; therefore, we assessed both traits with the RNAi lines and controls. In total, 24 (77%) of the tested RNAi knockdown alleles affected the mean and/or within line variance relative to the control: 21 genes affected the mean, 12 affected the variance, and nine affected both the mean and variance, suggesting at least partial joint regulation of mean and within line variance of food intake (Fig 4, S9 Table). The effects of RNAi knockdown alleles on the mean and variance of food intake were highly sex-specific, as expected from the quantitative genetic and GWA analyses, and elicited both increases and decreases in both traits, suggesting RNAi knockdown does not result in ‘sick’ flies. The functionally validated genes affecting mean and/or within line variance of food intake included octopaminergic genes [*Octopamine β3 receptor* (*Octβ3R*) and *Tyramine β hydroxylase* (*Tbh*)]; EGF signaling genes [*Epidermal growth factor receptor* (*Egfr*), *pointed* (*pnt*), *LDL receptor protein 1* (*LRP1*), and *CG7466*]; a hydrolase (*CG33226*); phosphatases [*Spn*, *twins* (*tws*), and *Protein tyrosine phosphatase 99A* (*Ptp99A*)]; and a calcium channel [*transient receptor potential* (*trp*)] (Fig 4, S9 Table, S10 Table).

## Discussion

### The genetic architecture of food consumption

There is substantial natural variation in food intake both within and between the sexes in the DGRP, ranging from the lowest intake in males from DGRP_307, which consume less than half a microliter over 24 hours, to the largest intake in females from DGRP_42, which consume more than four times that volume in the same time frame. The genetic architecture of mean food consumption from quantitative genetic analyses of feeding behavior in the DGRP, GWA analyses and functional validation studies is strikingly sex-specific. Perhaps surprisingly, natural variation in food intake is not highly correlated with organismal phenotypes related to body size and metabolism. Although there is a significant correlation with starvation resistance, less than 10% of the total phenotypic variation in food consumption can be explained by this association.

In addition to variation in mean food consumption, we show significant genetic variation in within-line environmental variance of food intake, adding to the growing list of quantitative traits for which there is genetic control of the magnitude of environmental variance [65–71]. We show for the first time in *Drosophila* that micro-environmental variance of food intake is heritable and that examining its genetic basis can yield insights into the regulation of both mean and micro-environmental variance of food intake. We tested 31 candidate genes from the GWA analyses for the mean and *CV*_*E*_ of food intake, and validated 24 (77%) of them. The validations were not always for the trait for which they were predicted by GWA. We speculate this is because the mean and variance of food consumption are correlated. Our functional validations were for the mean and variance; whereas the GWA was for the *CV*_*E*_. A common biological mechanism may underlie both feeding traits, which is not always true for the mean and micro-environmental plasticity of other *Drosophila* quantitative traits [92]. Note also that the direction, magnitude, and sex-specificity of the effects predicted by natural variants in the GWA analyses were not the same as for the effects of RNAi knockdown. This is not unexpected because the RNAi analyses test whether reducing gene expression affects feeding behavior, whereas the variants implicated by GWA may affect the trait via other mechanisms or may be associated with increased gene expression. In addition, the genetic background of the lines used for RNAi is different from the DGRP lines. Nevertheless, we can conclude from significant effects of RNAi knockdown on feeding behavior that these genes affect the traits when their function is compromised. In the future, more precise validations will be possible by CRISPR/Cas9 allelic replacements in the DGRP lines.

Previous studies examining the genetic underpinnings of food intake in *D*. *melanogaster* revealed interconnected sensory inputs, signaling pathways, and feedback loops integrated in the central nervous system [31–33, 35–45, 49–60, 93–105]. The GWA analyses for the mean and environmental variation of food intake complement these studies and identify many novel candidate genes. Two of the functionally validated genes affecting octopaminergic signaling, *Tbh* and *Octβ3R*, have been implicated previously to affect feeding behavior using different assays [83, 85, 86]; and a third functionally validated gene, *pnt*, has been implicated in feeding behavior by showing that mitochondrial biogenesis is influenced by larval nutrition [84]. The remaining functionally validated genes have no prior associations with food consumption.

### SNP functional validation and *CG18012*

We functionally validated a female-specific effect of *3R*_13637022_SNP on mean food intake. This potential regulatory polymorphism located upstream of *tinc* and *CG18012* could affect either gene. *tinc* encodes a transmembrane protein expressed in cardioblasts [106] and has no known mammalian orthologs [107]. *CG18012* was also validated to affect mean food consumption in males using RNAi. The human ortholog of *CG18012* is *Chitobiosyldiphosphodolichol beta-mannosyltransferase* (*ALG1*) (S10 Table) [107], which catalyzes the mannosylation step of lipid-linked oligosaccharide biosynthesis [108]. Mutations in *ALG1* can result in congenital defects of glycosylation, caused by deficient mannosyltransferase [109]. Characteristics of the congenital defect include abnormal adipose distributions and aberrant blood coagulation resulting in thromboses and hemorrhages [109]. Thus, *3R*_13637022_SNP most likely affects the gene to which it is most proximal, *CG18012*, although further work is necessary to understand the underlying mechanism. Note that the experimental design to test additive effects of SNPs implicated by GWA analyses in outbred DGRP genotypes can be generalized to any variant, including those in gene deserts. The observation that the SNP effect was female-specific and the RNAi-knockdown effect was male-specific is not unusual; different alleles of the same gene typically vary in the magnitude, direction and sex-specific effects on quantitative traits [reviewed in [110]].

### Bioamine signaling and feeding behavior

Two functionally validated genes from RNAi knockdown of gene expression were *Tyramine β hydroxylase* (*Tbh*) and *Octopamine β3 receptor* (*Octβ3R*). These genes both affect octopamine signaling. Tbh converts tyramine to octopamine [111]. *Tbh* is integral in the response to different sucrose concentrations and the habituation to sucrose in adult *D*. *melanogaster* and may affect carbohydrate metabolism since *Tbh* mutants are starvation resistant [86]. *Octβ3R* is a G-protein coupled octopamine receptor [112]. Mutations in *Octβ3R* affect post-starvation feeding in *Drosophila* larvae, suggesting *Octβ3R* is required for this response in fasted larvae [83]. The mammalian orthologs of *Octβ3R*, *histamine receptor H2* (HRH2) and *5-hydroxytryptamine receptor 4*, *G protein-coupled* (HTR4) and of *Tbh*, *dopamine beta-hydroxylase* (DBH) [107], function in human food ingestion and gastric emptying [113, 114] and rat feeding and glucoregulatory responses [115, 116] and post-prandial decreases in food intake [117], respectively. We functionally validated an effect of *CG34362* on food consumption using RNAi. The predicted mammalian ortholog of *CG34362* is the RNA metabolic gene *TIA1 granule-associated RNA binding protein* (*TIA1*). *TIA1* is down regulated in the fatty livers of obese humans and mice fed a high fat diet [118]. TIA1 functions in thyroid hormone regulation, and thyroid hormone signaling is integrated with adrenergic signaling in liver, adipose tissues, and the hypothalamus [reviewed in [119]]. Octopamine is the functional analog of mammalian norepinephrine. These results implicate an evolutionarily conserved pathway integrating metabolic information with bioamine signaling affecting feeding behavior.

### EGF signaling and food intake

We expected to find genes affecting insulin-like signaling in the GWA analyses, since aspects of *Drosophila* feeding are affected by components of the insulin-like signaling pathway [53, 58, 120, 121]. However, we did not observe this, possibly because these genes are under strong purifying selection and do not harbor functional variants at high enough frequencies to be detected by GWA analyses in the DGRP. We did, however, detect and functionally validated four components of EGF signaling, which is known to regulate insulin-like signaling and sensitivity [122–124]. EGF signaling is involved in sleep in *D*. *melanogaster* [125–127], but its role in *Drosophila* food intake has not been demonstrated previously. We show from our RNAi functional validation that the EGF receptor, *Egfr*, affects *Drosophila* feeding behavior. The mammalian EGFR and components of the mammalian EGF signaling pathway are known to enhance glucose absorption in the gut [128], induce obesity in ovariectomized [129] and aged female mice [130], are decreased in adipose tissues of calorie restricted mice [131], and are associated with hyperglycemia in humans [132]. Lipid Transfer Protein (LTP) is produced by the *Drosophila* fat body and conveys information about dietary lipid composition to specific neurons innervating insulin-like-producing cells and thereby affecting insulin signaling [124]. *LRP1*, which is a promiscuous receptor in EGF signaling, allows LTP to cross the *Drosophila* blood brain barrier to affect insulin-like signaling [124]. Mammalian *LRP1* regulates leptin signaling and energy homeostasis [133], modulates cholesterol metabolism [134], controls adipose tissue generation [135] in the development of obesity [136] with obese adipose tissue upregulation [135, 137], is associated with blood biomarkers of diet-based obesity interventions [138], controls post ingestion lipid transport and glucose regulation [139], and correlates dietary carbohydrate metabolism to metabolic syndrome [140] and fatty acid intake with body mass index [141–143].

The EGF signaling gene *pnt* encodes an ETS-1 transcription factor; ETS1 and ETS2 orthologs in mammals [107] are activated by EGF signaling [144, 145]. In *Drosophila*, persistent EGF signaling via *pnt* induces insulin resistance, downregulating expression of the *Insulin-like receptor* gene [123]. Further, *pnt* gene expression is correlated with larval nutrient control of mitochondrial biogenesis [84]. The mammalian orthologs are increased in the serum of obese humans [146], correlate with adipose tissue generation [147], increase obesity-related adipose tissue inflammation [148], are upregulated in diabetes [149], act in the development of diabetic retinopathy [150] and nephropathy [151], and reduce endothelial progenitor cells in diabetes [152]. While the mammalian ortholog of the EGF signaling gene *CG7466*, *multiple EGF-like-domains 8* (*MEGF8*) [107], did not have a role in feeding or metabolically related mammalian traits, we did functionally validate it in *Drosophila*. Given the variety of metabolically related mammalian phenotypes these EGF signaling genes affect without directly affecting food consumption, we posit *Drosophila* as an essential tool in elucidating the roles of EGF signaling genes in directly regulating feeding behavior.

### Novel insights in the genetic basis of feeding behavior

Eight of the 24 genes validated using RNAi either have mammalian orthologs with no known function in metabolically related phenotypes (*CG2121*, *mitochondrial ribosomal protein L54* (*mRpL54*), *tropomodulin* (*tmod*), *Ptp99A*, and *CG7466*) or have no known mammalian orthologs (*CG34356*, and *Gag related protein* (*Grp*)) [107]. The remaining genes have mammalian orthologs involved in the development of various metabolically related phenotypes and diseases. The orthologs, however, typically do not affect mammalian food intake directly but rather affect the etiology of these complex phenotypes. Future analyses of the mechanisms by which these genes affect *Drosophila* feeding harbors the potential to better elucidate the means by which various mammalian metabolic phenotypes are also regulated.

*branchless* (*bnl*) affects variance of food intake in males. *bnl* encodes a growth factor that interacts with hedgehog signaling in a variety of developmental processes [153–155]. Hedgehog signaling controls fat development in *Drosophila* [156, 157] and mice [156] and coordinates fat development with nutrients [158]. The mammalian orthologs of *bnl* include a variety of fibroblast growth factors (FGFs) (S10 Table) [107]. Treatment with FGF1 restores normal blood sugar levels in a murine model of type 2 diabetes [159], and FGF1 knockout mice develop aggressive type 2 diabetes and aberrant adipose expansion [160]. Additionally, FGF1 is critical for energy homeostasis, and FGF10 plays an essential role in the development of white adipose pads [reviewed in [161]].

*CG33226* is nested within *Egfr*. *CG33226* encodes a predicted serine protease of which *Granzyme B* (*GzmB*), an immune system enzyme, is the mammalian ortholog [107]. *GzmB* increases inflammation in type 1 and type 2 diabetes [162] and impairs insulin secretion from islet cells [163] as part of the mechanism by which it kills pancreatic β cells in the development of type 1 diabetes [162, 164].

RNAi against *LysP*, which encodes the immune system enzyme glycoside hydrolase, affects mean food consumption in males and females. A mammalian ortholog of *LysP* is *Lysozyme* (*Lyz*) [107]. In mammals, high LYZ protein levels negatively correlate with decreased Paneth intestinal cells observed in the development of obesity [165, 166], and *Lyz* vascular transcript levels are upregulated in athersclerotic humans and obese juvenile swine [167]. RNAi of *lilliputian* (*lilli*) affects mean food consumption in females. The human ortholog of *lilli* is *AF4/FMR2 family*, *member 1* (AFF1) [107], an adipogenic transcription factor that is also upregulated in juvenile obese adipose tissue [168].

We functionally validated *Spn*, *tws*, and *Ptp99A* to affect *Drosophila* feeding. The mammalian orthologs of these genes are protein phosphatases (S10 Table). The orthologs of *Spn*, *protein phosphatase 1*, *regulatory subunit 9A* (*PPP1R9A*) and *protein phosphatase 1*, *regulatory subunit 9B* (*PPP1R9B*), are associated with piglet birth weight [169] and pig food intake and human obesity [170]. The mammalian orthologs of *tws*, *protein phosphatase 2*, *regulatory subunit B*, *alpha* and *delta* (*PPP2R2A* and *PPP2R2D*) [107] dephosphorylate FOXO1 to cause its nuclear import during the oxidative stress accompanying pancreatic β cell death of diabetes [171], associate with increased lipid levels upon high fat feeding [172], and are regulated by microRNA-136 in the aberrant proliferation of vascular smooth muscle cells that accompanies atherosclerosis [173]. The orthologs of *Ptp99A* are not known to function in mammalian feeding or metabolically related phenotypes, but the novel elucidation of these phosphatases affecting feeding in *Drosophila* supports further *Drosophila*-based work to understand the evolutionarily conserved mechanisms by which they act in food intake to better understand how they act in other metabolically related traits.

RNAi of *slamdance* (*sda*) affects the mean and variance of food intake in females. The mammalian *sda* ortholog, *alanyl aminopeptidase* (*ANPEP*), is a receptor metalloproteinase. *ANPEP* has been associated with regulating networks involved in the development of type 2 diabetes [174] and may underlie pancreatic β cell fate during metabolic stress [175]. RNAi of *Limpet* (*Lmpt*) affects females food intake. The *Lmpt* mammalian ortholog is *four and a half LIM domains 2* (*FHL2*) [107], which has been reported to activate Wnt signaling in the pathogenesis of diabetic nephropathy [176].

Transient receptor potential (TRP) channels are ion channels mediating a variety of sensations. In *Drosophila*, TRPL underlies long-term desensitization to bitter food compounds [32] and *painless*, the *Drosophila* TRPA channel, mediates larval post-feeding migration away from food [177] and avoidance of pungent tastants [178]. Here we demonstrate that *trp*, the *Drosophila* TRPC channel [107], affects total food intake in females. In mammals, TRPC channels play an important role in platelet function [179–181], affecting thrombosis and platelet aggregation [181]. *TRPC3* is upregulated in platelets of type 2 diabetics [182]. TRPC channels at least partially underlie diabetic complications including cardiovascular comorbid pathophysiology via vascular tissue signaling pathway perturbation [183–185] and diabetic nephropathy [186–188].

## Conclusions

We have utilized natural variation in the mean and within line variance of food consumption in the DGRP [63, 64] to identify and functionally validate novel genes and a SNP affecting food intake. Most of these genes have mammalian orthologs that are implicated in the development of many metabolically related diseases, such as type 2 diabetes, while not generally affecting food intake volume directly. These results position *D*. *melanogaster* as a unique model to elucidate the etiology of these complex phenotypes. Future work to address the tissue specificity of where and how these genes act and interact will shed further light on the extraordinary sexual dimorphism and the genetic bases of food intake and variation of micro-environmental plasticity of food intake. RNAi suppression of 17 of the confirmed candidate genes affecting mean food intake led to an increase in mean food consumption, suggesting that these genes normally act to limit food intake and that many more peptides, genes, and signaling pathways are involved in the attenuation of food intake than in its initiation or continuation [38–50]. The genetic regulation of *D*. *melanogaster* food intake behavior is similar to that of humans, further establishing this model as an integral tool for understanding why individuals eat different amounts of food.

## Materials and Methods

### *Drosophila* stocks and culture

We used 182 sequenced, wild-derived, inbred DGRP lines (S1 Table) to perform GWA analyses. We obtained RNAi transgenic fly lines (*bnl* ^101377^, *CG10477* ^103490^, *CG18012* ^20580^, *CG2121* ^109845^, *CG32107* ^102854^, *CG33226* ^102384^, *CG34356* ^109790^, *CG34362* ^107503^, *CG7466* ^42462^, *CG7920* ^21577^, *Dop1R2* ^105324^, *Dys* ^108006^, *Egfr* ^107130^, *Grp* ^110076^, *lilli* ^106142^, *Lmpt* ^100716^, *LpR2* ^107597^, *LRP1* ^109605^, *LysP* ^110747^, *Mp* ^107319^, *mRpL54* ^105729^, *Octβ3R* ^101189^, *pnt* ^105390^, *Ptp99A* ^103931^, *sda* ^100215^, *Spn* ^105888^, *Tbh* ^107070^, *tmod* ^108389^, *trp* ^1365^, *tws* ^104167^, *yip7* ^102226^) and the corresponding progenitor lines (60000 and 60010) from the Vienna *Drosophila* RNAi Center (VDRC) [189]. We crossed these lines to a weak, ubiquitously expressed driver, and we crossed the driver to the progenitor RNAi controls (v60000 and v60010) to serve as our experimental controls. To generate the driver, we obtained the *Ubiquitin-GAL4* stock from the Bloomington *Drosophila* Stock Center. We replaced the major chromosomes with isogenic *w* ^*1118*^; *Canton-S-B* [190] chromosomes, except the X chromosome and chromosome 2 with the driver construct. A new driver stock, *Ubiquitin-GAL4[156]*, was created by introducing the original *Ubi-GAL4* transgene onto the *Canton-S-B* third chromosome using *Δ2–3* mediated hopping. The *X* and second chromosome were then replaced with *Canton-S-B* chromosomes. The driver stock was generated and provided by Dr. Akihiko Yamamoto. All lines were reared in small mass cultures on cornmeal/molasses/agar medium under standard culture conditions (25°C, 12:12 hour light/dark cycle).

### Behavior assays

We used mated 3–7 day old flies in all assays. After an 18-hour period of food deprivation on 1.5% agar, we placed groups of 8 flies from the same sex and genotype in individual vials containing 2 mL 1.5% agar medium and three 5 μL capillary tubes (Kimble Glass Inc.) containing a 4% sucrose solution inserted through a foam plug. Our modified version of the CAFE assay [24] includes the agar medium to prevent desiccation affecting food consumption. We placed the CAFE vials in a transparent plastic container in which high humidity is maintained with open containers of water to minimize evaporation from the capillary tubes. In addition, we assessed evaporation by placing CAFE vials with capillary tubes containing 4% sucrose but without flies in the same humid chamber. We measured total food consumption in each CAFE vial after 24 hours, between 10 A.M. and 12 P.M. We adjusted the total amount of food consumed by the average evaporation that occurred in the control vials and the total number of flies alive at the end of the assay. We performed six replicate assays per sex and DGRP line, and 10–12 replicates per sex and genotype for RNAi knock down and their respective controls and SNP-based crosses.

### Quantitative genetics of mean food consumption in the DGRP

We used a linear mixed model to partition variance in mean food consumption across DGRP lines and between sexes with the following model: *Y* = *μ* + *L* + *S* + *L*x*S* + *E*, where *Y* is the phenotype, *L* is the random main effect of line, *S* is the fixed main effect of sex, and *E* is the error term. We also fitted reduced models of form *Y* = *μ* + *L* + *E* for each sex separately. We estimated the broad-sense heritability (*H* ^2^) pooled across sexes as *H* ^2^ = (*σ* ^2^_*L*_ + *σ* ^2^_*SL*_)/ (*σ* ^2^_*L*_ + *σ* ^2^_*SL*_ + *σ* ^2^_*E*_), where *σ* ^2^_*L*_, *σ* ^2^_*SL*_, and *σ* ^2^_*E*_ are the among-line, sex by line and within-line variance components, respectively. We estimated broad sense heritabilities for each sex as *H* ^2^ = *σ* ^2^_*L*_/(*σ* ^2^_*L*_ + *σ* ^2^_*E*_). We estimated the cross-sex genetic correlation as *r*_*GS*_ = *cov*_*MF*_ */σ*_*LM*_ *σ*_*LF*_, where *cov*_*MF*_ is the covariance of line means between males and females, and *σ*_*LM*_ and *σ*_*LF*_ are the among line standard deviations for males and females.

### Associations of food intake with other quantitative traits in the DGRP

We computed Pearson’s product moment correlations of line means for food consumption and other quantitative traits previously measured on the DGRP lines.

### Quantitative genetics of within-line variation in food consumption in the DGRP

We assessed whether there was heterogeneity of within-line environmental variance in food consumption among the DGRP lines, using both Brown-Forsythe and Levene’s tests [65]. We used the coefficient of environmental variation for each line (*CV*_*E*_ = 100*σ*_*E*_/*mean*), where *σ*_*E*_ is the standard deviation of food consumption between replicate assays with each line and *mean* is the line mean, as our metric of within line environmental variance, to partially account for any dependence of the variance on mean food consumption.

### GWA analyses for food consumption

We performed GWA analyses using the 1,920,276 SNPs and indels with minor allele frequencies ≥ 0.05 on the DGRP webserver [64] (dgrp2.gnets.ncsu.edu). We performed four analyses each for the mean and for the *CV*_*E*_ of food consumption: the average of females and males, the difference between females and males (for assessing sexual dimorphism in the QTL effects), and females and males separately. These GWA analyses account for effects of *Wolbachia* infection, cryptic relatedness due to major inversions, and residual polygenic relatedness [64]. We performed gene ontology enrichment and network analyses based on the top (*P* < 10^−5^) variants associated with the mean of food consumption using DAVID bioinformatics [87, 88].

### Functional validation

We functionally tested genes with top associations using RNAi knock down alleles and their respective control lines. We assessed whether differences in mean food intake between RNAi knock down alleles and the controls were significant using Dunnett’s tests (JMP 10, SAS), separately for males and females. We also assessed significant variation in environmental variance between RNAi knock down alleles and the controls with pairwise Levene’s tests (JMP 10, SAS), separately for males and females.

We performed one SNP-based test (*3R*_13637022_SNP near *tinc* and *CG18012*) using the DGRP lines. We randomly selected ten minor-allele containing DGRP lines and ten major-allele containing DGRP lines. Within each genotype class, we randomly selected five DGRP lines to be the male parent and five to be the female parent, thus producing 10 F_1_ genotypes that are homozygous for either the focal major or minor alleles and outbred elsewhere. We assessed mean food consumption with 10–12 replicates per sex per F_1_ genotype, and used *t*-tests to evaluate significant differences between the major and minor alleles, separately for males and females.

## Supporting Information

S1 Fig Association between the number of segregating sites in the DGRP and *CV*_*E*_ of food intake. Red diamonds: females. Blue squares: males.
(PDF)

S1 Table Food consumption in the DGRP. (**A**) Raw data. (**B**) Summary statistics.
(XLSX)

S2 Table Phenotypic correlations (*r*) between food intake and other organismal phenotypes in the DGRP. Cells highlighted in yellow depict significant (*P* < 0.05) correlations. Phenotypic values from the same sources have the same color codes.
(XLSX)

S3 Table ANOVA of the effects of *Wolbachia* infection and common polymorphic inversions on mean and *CV*_*E*_ of food intake. df: degrees of freedom; SS: Type III sums of squares; F: F statistic; AIC: Akaike information criterion. ***: *P* < 0.001; **: *P* < 0.01; *: *P* < 0.05.
(DOCX)

S4 Table Top (nominal *P* < 10^−5^) variants associated with mean food intake. (XLSX)

S5 Table Top (nominal *P* < 10^−5^) variants associated with *CV*_*E*_ of food intake. (XLSX)

S6 Table Candidate genes affecting mean and *CV*_*E*_ of food intake. Previous associations with feeding related traits are given.
(XLSX)

S7 Table Gene Ontology enrichment analyses for mean and *CV*_*E*_ of food intake. Only nominally enriched GO categories were observed.
(XLSX)

S8 Table Functional Validation for *3R*:13637022_SNP. (**A**) Summary statistics. (**B**) Raw data. All units are *μ*L/fly. M: male parent; F: female parent.
(XLSX)

S9 Table RNAi functional validation. (**A**) *P*-values from tests of differences from controls. (**B**) Raw data. (**C**) Summary statistics.
(XLSX)

S10 Table Summary of genes validated by RNAi knock down. (XLSX)
