# Supplementary figures and images for: Quantitative Genetics of Food Intake in Drosophila melanogaster
Source: PLoS One. 2015 Sep 16;10(9):e0138129. doi: 10.1371/journal.pone.0138129 (PMC4574202; doi:10.1371/journal.pone.0138129)

**S1 Figure.** Association between the number of segregating sites in the DGRP and  $CV_E$  of food intake.

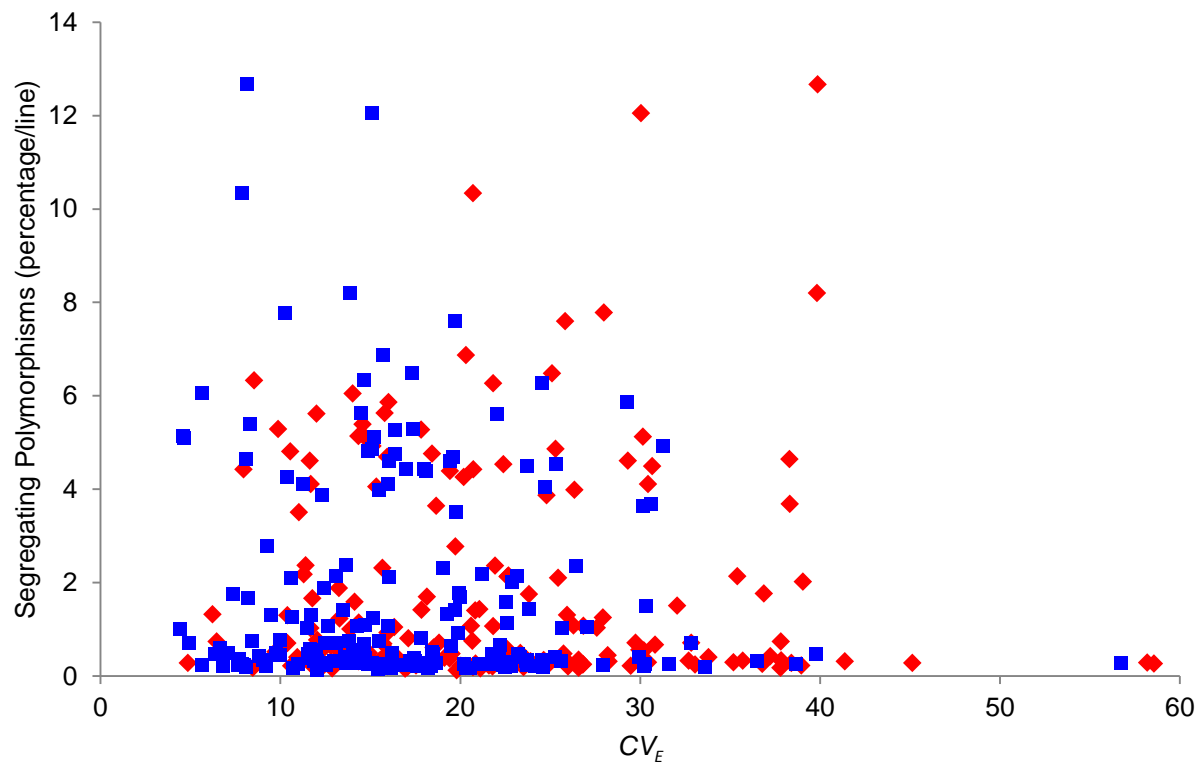

Supplement: S1 Fig — Red diamonds: females. Blue squares: males. (PDF) [file pone.0138129.s001.pdf]
