# Supplementary material for: Quantitative Genetics of Food Intake in Drosophila melanogaster
Source: PLoS One. 2015 Sep 16;10(9):e0138129. doi: 10.1371/journal.pone.0138129 (PMC4574202; doi:10.1371/journal.pone.0138129)
Supplement: S3 Table — df: degrees of freedom; SS: Type III sums of squares; F: F statistic; AIC: Akaike information criterion. ***: P < 0.001; **: P < 0.01; *: P < 0.05. (DOCX) [file pone.0138129.s004.docx]

**S3 Table.** **ANOVA of the effects of *Wolbachia* infection and common polymorphic inversions on mean and CVE of food intake**. df: degrees of freedom; SS: Type III sums of squares; F: F statistic; AIC: Akaike information criterion. ***: *P* < 0.001; **: *P* < 0.01; *: *P* < 0.05.

| Analysis | Covariates | df | SS | Residual SS | AIC | F | *P*-value |
| --- | --- | --- | --- | --- | --- | --- | --- |
| Female Mean | none |  |  | 2338.8 | 488.72 |  |  |
|  | *Wolbachia* | 1 | 62.45 | 2401.3 | 491.52 | 4.54 | 0.03* |
|  | *In(2L)t* | 2 | 5.54 | 2344.4 | 485.15 | 0.20 | 0.82 |
|  | *In(2R)NS* | 2 | 16.41 | 2355.3 | 485.99 | 0.60 | 0.55 |
|  | *In(3R)P* | 2 | 25.61 | 2364.5 | 486.7 | 0.93 | 0.40 |
|  | *In(3R)K* | 2 | 27.21 | 2366.1 | 486.82 | 0.99 | 0.37 |
|  | *In(3R)Mo* | 2 | 69.17 | 2408 | 490.02 | 2.51 | 0.08 |
| Male Mean | none |  |  | 1706.8 | 430.14 |  |  |
|  | *Wolbachia* | 1 | 2.60 | 1709.4 | 428.42 | 0.26 | 0.61 |
|  | *In(2L)t* | 2 | 9.10 | 1715.9 | 427.11 | 0.45 | 0.64 |
|  | *In(2R)NS* | 2 | 10.25 | 1717.1 | 427.23 | 0.51 | 0.60 |
|  | *In(3R)P* | 2 | 3.33 | 1710.2 | 426.5 | 0.16 | 0.85 |
|  | *In(3R)K* | 2 | 62.85 | 1769.7 | 432.69 | 3.11 | 0.05* |
|  | *In(3R)Mo* | 2 | 65.13 | 1772 | 432.92 | 3.22 | 0.04* |
| Sex  Average  Mean | none |  |  | 1682.1 | 427.5 |  |  |
|  | *Wolbachia* | 1 | 22.85 | 1704.9 | 427.94 | 2.30 | 0.13 |
|  | *In(2L)t* | 2 | 4.72 | 1686.8 | 424.01 | 0.24 | 0.79 |
|  | *In(2R)NS* | 2 | 13.18 | 1695.2 | 424.91 | 0.66 | 0.52 |
|  | *In(3R)P* | 2 | 11.37 | 1693.4 | 424.72 | 0.57 | 0.57 |
|  | *In(3R)K* | 2 | 40.70 | 1722.8 | 427.83 | 2.04 | 0.13 |
| Sex  Difference  Mean | none |  |  | 1345.4 | 387.07 |  |  |
|  | *Wolbachia* | 1 | 40.16 | 1385.5 | 390.40 | 5.05 | 0.03* |
|  | *In(2L)t* | 2 | 13.15 | 1358.5 | 384.83 | 0.83 | 0.44 |
|  | *In(2R)NS* | 2 | 0.74 | 1346.1 | 383.17 | 0.05 | 0.95 |
|  | *In(3R)P* | 2 | 10.43 | 1355.8 | 384.47 | 0.66 | 0.52 |
|  | *In(3R)K* | 2 | 17.17 | 1362.5 | 385.37 | 1.08 | 0.34 |
|  | *In(3R)Mo* | 2 | 2.14 | 1347.5 | 383.36 | 0.13 | 0.87 |
| Female *CV_E_* | none |  |  | 14764 | 820.67 |  |  |
|  | *Wolbachia* | 1 | 6.66 | 14771 | 818.75 | 0.08 | 0.78 |
|  | *In(2L)t* | 2 | 186.92 | 14951 | 818.94 | 1.07 | 0.35 |
|  | *In(2R)NS* | 2 | 291.67 | 15056 | 820.21 | 1.67 | 0.19 |
|  | *In(3R)P* | 2 | 52.59 | 14817 | 817.31 | 0.30 | 0.74 |
|  | *In(3R)K* | 2 | 317.64 | 15082 | 820.52 | 1.82 | 0.17 |
|  | *In(3R)Mo* | 2 | 50.23 | 14814 | 817.28 | 0.29 | 0.75 |
| Male  *CV_E_* | none |  |  | 9798.3 | 746.46 |  |  |
|  | *Wolbachia* | 1 | 1.486 | 9799.8 | 744.48 | 0.03 | 0.87 |
|  | *In(2L)t* | 2 | 80.86 | 9879.1 | 743.94 | 0.70 | 0.50 |
|  | *In(2R)NS* | 2 | 267.98 | 10066 | 747.34 | 2.31 | 0.10 |
|  | *In(3R)P* | 2 | 85.84 | 9884.1 | 744.03 | 0.74 | 0.48 |
|  | *In(3R)K* | 2 | 241.16 | 10039 | 746.86 | 2.08 | 0.13 |
|  | *In(3R)Mo* | 2 | 270.74 | 10069 | 747.39 | 2.33 | 0.10 |
|  |  |  |  | 7083.9 | 687.74 |  |  |
| Sex  Average *CV_E_* | none |  | 3.61 | 7087.5 | 685.83 | 0.09 | 0.77 |
|  | *Wolbachia* | 1 | 93.13 | 7177 | 686.11 | 1.11 | 0.33 |
|  | *In(2L)t* | 2 | 170.50 | 7254.4 | 688.05 | 2.03 | 0.13 |
|  | *In(2R)NS* | 2 | 10.51 | 7094.4 | 684.01 | 0.13 | 0.88 |
|  | *In(3R)P* | 2 | 104.86 | 7188.7 | 686.40 | 1.25 | 0.29 |
|  | *In(3R)K* | 2 | 137.28 | 7221.2 | 687.22 | 1.64 | 0.20 |
|  | *In(3R)Mo* | 2 |  | 7083.9 | 687.74 |  |  |
| Sex  Difference  *CV_E_* | none |  |  | 20790 | 882.61 |  |  |
|  | *Wolbachia* | 1 | 1.85 | 20791 | 880.63 | 0.02 | 0.90 |
|  | *In(2L)t* | 2 | 163.04 | 20953 | 880.03 | 0.66 | 0.52 |
|  | *In(2R)NS* | 2 | 437.29 | 21227 | 882.38 | 1.78 | 0.17 |
|  | *In(3R)P* | 2 | 234.85 | 21024 | 880.64 | 0.95 | 0.39 |
|  | *In(3R)K* | 2 | 698.15 | 21488 | 884.59 | 2.84 | 0.06 |
|  | *In(3R)Mo* | 2 | 92.85 | 20882 | 879.42 | 0.38 | 0.69 |
